# Effects of mesenchymal stem cells in renovascular disease of preclinical and clinical studies: a systematic review and meta-analysis

**DOI:** 10.1038/s41598-022-23059-2

**Published:** 2022-10-27

**Authors:** Hong-Shen Wang, Ming-Yu Yi, Xi Wu, Qian Liu, Ying-Hao Deng, Ting Wu, Lin Wang, Yi-Xin Kang, Xiao-Qin Luo, Ping Yan, Mei Wang, Shao-Bin Duan

**Affiliations:** 1 Department of Nephrology, The Second Xiangya Hospital of Central South University, Hunan Key Laboratory of Kidney Disease and Blood Purification, 139 Renmin Road, Changsha, 410011 Hunan China; 2 Department of Anesthesiology, The Third Xiangya Hospital of Central South University, Changsha, Hunan China

**Keywords:** Stem cells, Mesenchymal stem cells, Kidney diseases, Renal artery stenosis

## Abstract

Renal artery stenosis (RAS) causes severe renovascular hypertension, worsening kidney function, and increased cardiovascular morbidity. According to recent studies, mesenchymal stem cells (MSCs) administration is a promising therapy for the improvement of RAS outcomes. The meta-analysis aims to evaluate the therapeutic effects of MSC therapy on RAS. We performed a search in MEDLINE, Web of Science, Embase, and Cochrane Library from inception to 5, October 2022. We included 16 preclinical and 3 clinical studies in this meta-analysis. In preclinical studies, the pooled results indicated that animals treated with MSCs had lower levels of systolic blood pressure (SBP) (SMD = − 1.019, 95% CI − 1.434 to − 0.604, I^2^ = 37.2%, P = 0.000), serum creatinine (Scr) (SMD = − 1.112, 95% CI − 1.932 to − 0.293, I^2^ = 72.0%, P = 0.008), and plasma renin activity (PRA) (SMD = − 0.477, 95% CI − 0.913 to 0.042, I^2^ = 43.4%, P = 0.032). The studies also revealed increased levels of renal blood flow (RBF) in stenotic kidney (STK) (SMD = 0.774, 95% CI − 0.351 to 1.197, I^2^ = 0%, P = 0.000) and the glomerular filtration rate (GFR) of STK (SMD = 1.825, 95% CI 0.963 to 2.688, I^2^ = 72.6%, P = 0.000). In clinical studies, the cortical perfusion and fractional hypoxia of the contralateral kidney (CLK) were alleviated by MSC therapy. Taken together, this meta-analysis revealed that MSCs therapy might be a promising treatment for RAS. However, due to the discrepancy between preclinical studies and early clinical trials outcomes, MSC therapy couldn’t be recommended in clinical care for the moment, more high-quality randomized controlled clinical trials are needed to validate our conclusions and standardize MSCs protocols.

## Introduction

Renal artery stenosis (RAS) is the primary cause of renovascular hypertension (RVH)^1^. RAS prevalence is approximately 1–3% of the population with hypertension and up to 20% of the population with secondary hypertension^2–4^. Kwon et al. showed that RAS occurred more frequently in hypertensive patients aged 65 years or older^5^. RAS increases hypertension and post-stenotic kidney damage^6^, leading to end-stage renal disease, more specifically in elderly patients. What’s more, patients with RAS have an increased risk for developing cardiovascular complications, increasing morbidity and mortality^7^. Conlon et al. reported that the presence of RAS in patients with coronary disease independently doubled the risk of mortality even when coronary revascularisation was performed^8^. In past decades, lipid lowering-drugs, antihypertensive drugs, and renal revascularization were used to treat RAS. However, two large clinical trials, ASTRAL and CORAL, demonstrated that renal revascularization had no advantage when compared to the medical treatment^9–11^. This might because not merely chronic hypoxia and reduced blood flow, but activation of the renin–angiotensin–aldosterone system, increased oxidative stress and cytokine release, microvascular dysfunction and rarefaction, and kidney fibrosis are involved in the underlying mechanisms. Conversely, recommendations of therapy were discordant in various guidelines. RAS patients suffering irreversible loss of kidney function and persistent hypertension usually develop into end-stage kidney disease (ESRD) and cardiovascular events, causing heavy economic burden for families and society, especially in developing countries. Therefore, it is necessary to develop a more effective alternative or adjunctive therapy for RAS treatment.

Cell-based therapies attracted attention in numerous fields of medical research in recent years. Mesenchymal stromal cells (MSCs) are isolated from various adult tissues, such as bone marrow, adipose tissue, umbilical cord blood, and deciduous teeth^12^. Due to the capacity of their differentiation potential, self-renewal and immunomodulatory cytokine secretion, MSCs was widely studied in many diseases, including autoimmune disorders^13^, acute kidney injury^14^, chronic renal failure^15^, diabetic kidney disease^16^, and stroke^17^.

To date, some studies regarding RAS indicate that the administration of MSCs can ameliorate the loss of renal blood flow, kidney function, as well as elevate the release of inflammatory factors. However, the efficacy of MSCs administration on RAS remains unclear. There is a reinforced need to conduct an overview that patients and clinicians can utilize. Thus, this systematic review and meta-analysis of preclinical and clinical studies aims to evaluate the efficacy of the MSCs treatment in RAS.

## Materials and methods

### Search strategy

We searched four online databases: MEDLINE, Embase, Cochrane Library, and Web of Science from up to October 5, 2022. Searched terms used in this article were listed as follows: (mesenchymal stem cells OR mesenchymal stromal cells OR multipotent stromal cells OR mesenchymal progenitor cells) AND (renovascular disease OR Renal artery stenosis OR renal Artery Obstruction OR RAS OR RVD). Furthermore, reference lists were also reviewed for the possibility of additional literature. Two researchers independently screened the collected articles.

### Eligibility criteria

Inclusion criteria of the eligible literature are as follows: (1) randomized controlled trials, comparative studies, or controlled trials involving animal models of RAS or patients with RAS, (2) animals or patients in the studies who received MSCs treatment, (3) studies that have a corresponding comparison group, (4) Data regarding MSC treatment or control groups should be provided in detail, (5) studies that provided efficacy outcomes (e.g., blood pressure, blood flow, and serum creatinine).

Exclusion criteria are as follows: (1) case reports, meeting abstracts, repeat studies, letters, reviews, or meta-analysis and studies where the full text was unavailable, (2) studies with insufficient data, (3) studies that focused on the treatment using other stem cells or agents.

### Study selection and data extraction

Two investigators independently reviewed the titles and abstracts according to inclusion and exclusion criteria. Once disagreements arose, a third investigator reviewed the articles and made the decision. Relevant data were recorded in a standardized form in Microsoft Excel. The preclinical form included the first author, year, location, species, the number of groups, type of MSC, modeling methods, the dosage of MSC, delivery, follow-up duration, and efficacy indicators (e.g., systolic blood pressure, degree of stenosis, glomerular filtration rate (GFR), renal blood flow, serum creatinine (Scr), urine protein, body weight, and inflammatory markers). The clinical form included the first author, year, location, number of groups, type of MSC, modeling methods, the dosage of MSC, route of MSC delivery, endpoints, and follow-up duration. The data were extracted from graphics using Get Data Graph Digitizer 2.25 software for studies that did not supply direct results.

### Quality assessment

For preclinical studies, the Systematic Review Centre for Laboratory animal Experimentation (SYRCLE) risk of bias tool was employed for quality assessment^18^. For the clinical studies, we used the Methodological Index For Non-randomized Studies (MINORS) tool for the non-randomized controlled studies^19^. The GRADE assessment was also performed to assess the quality of the evidence. The quality assessment was carried out by two investigators independently, and a third investigator resolved any disagreements.

### Statistical analysis

This study followed the recommended PRISMA statement. STATA 12.0 statistical software package (Stata Corporation, College Station, TX) was used for statistical analysis. All median with range or interquartile range were converted to the form mean with standard deviation^20^. The weighted mean difference (WMD) and standard mean difference (SMD) with 95% confidence intervals (CIs) were used for appropriate continuous variables. According to previous studies, we used the method below to choose the effect model. The effects of the outcomes were pooled using a fixed-effect model, while a random model was employed when significant heterogeneity was detected. Heterogeneity was assessed by I^2^ and considered significant when I^2^ > 50%^21–23^. Potential publication bias was assessed via Funnel plots, as well as the Bagger’s and Egger’s tests. P < 0.05 (two-sided) was considered statistically significant in our meta-analysis.

## Results

### Study selection

**Figure 1:**
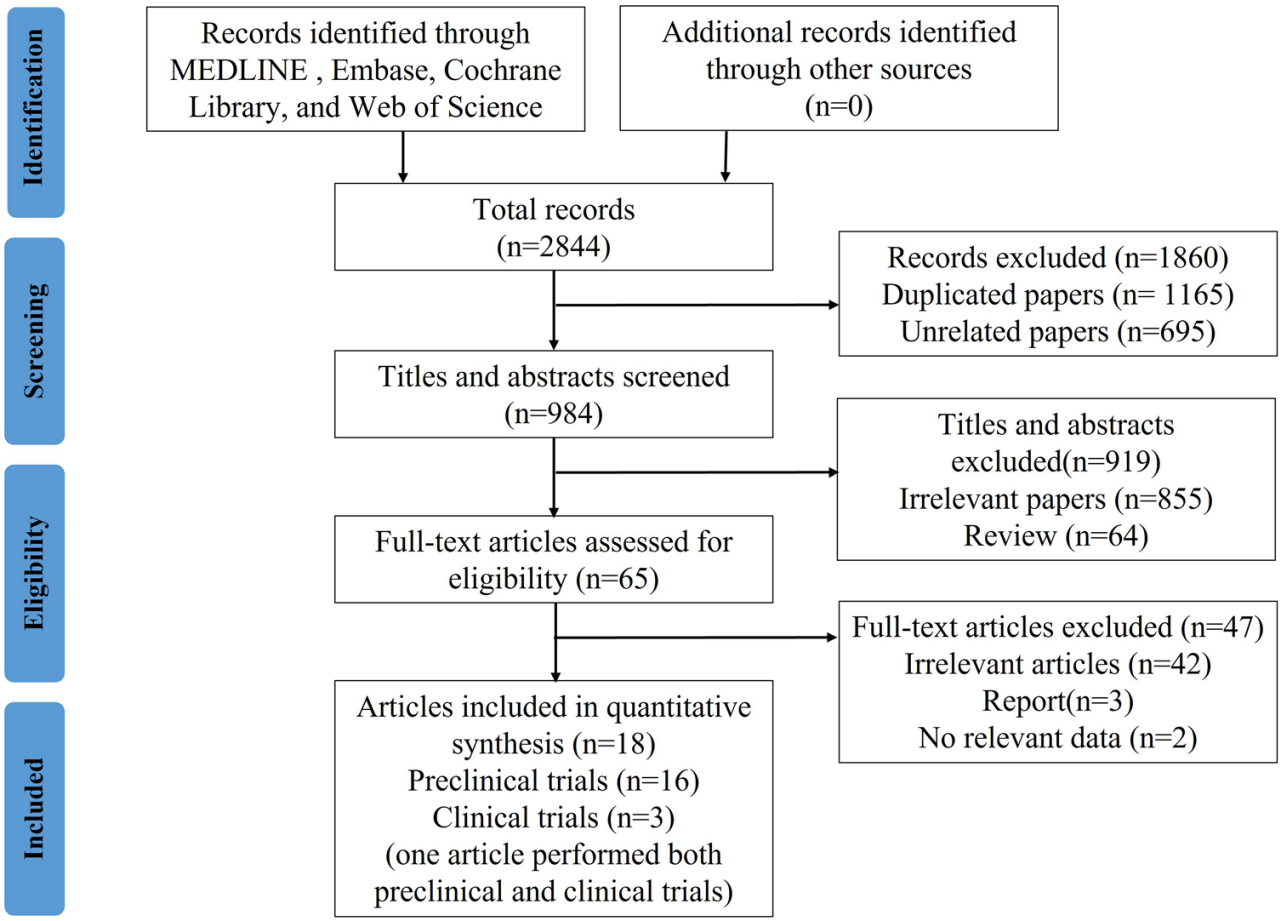
Flow chart of study selection.

Our study identified 2844 relevant studies initially, including clinical and preclinical studies. After removing the duplicates and screening the titles and abstracts, 65 studies were left. The remaining 65 studies were carefully reviewed and 47 were further excluded due to lack of data, being off-topic, and unavailability of full texts. The flowchart for the screening process of the eligible trials is shown in Fig. 1. In brief, 18 articles involving 16 preclinical^24–39^ and 3 clinical studies^31,40,41^ were included in our meta-analysis, in which one study performed both animals and human trials. If more than one experiment was carried out in a single study, we regarded each experiment as independent.

### Study characteristics

In 16 preclinical studies, 8 were conducted in pig models, 6 were conducted in rat models, and 2 were conducted in mouse models. The RAS or RVH model was induced using an irritant coil placed in one main renal artery. Additionally, a high-cholesterol diet was used in the atherosclerotic renal artery stenosis (ARAS) models. Different types of MSCs were used within the studies, adipose-derived stem cells (ADSCs) (n = 11) and bone marrow mesenchymal stem cells (BM-MSCs) (n = 5) were included. The characteristics of the included animal studies are listed in Table 1.

**Table 1:** Characteristics of preclinical studies.

	Author	Year	Country	Species	Study design	Treatment group	Control group	Model features	MSC source	MSCs dose	Route	Endpoints	Duration
1	Eirin et al.	2015	USA	Pig	Unclear	7	7	An irritant coil was placed in the main renal artery under fluoroscopy	AD-MSCs	1.0 × 10^7^	MSCs were injected slowly through a balloon catheter placed in the renal artery proximal to the stenosis	Body weight, degree of stenosis, mean arterial pressure, IFN-γ, TNF-α, IL-10, MCP-1, GFR, RBF and kidney histopathology	10 weeks
2	Zhu et al.(A)	2013	USA	Pig	Unclear	6	7	An irritant coil was placed in one renal artery under anesthesia	AD-MSCs	1.0 × 10^7^	MSCs were injected slowly through a balloon placed in the renal artery proximal to the stenosis	RBF and GFR, microvascular density, kidney histopathology, Scr, urine protein, body weight, degree of stenosis, mean arterial pressure, TNF-α, IL-10	10 weeks
3	Zhu et al.(B)	2013	USA	Pig	Unclear	3	6	An irritant coil was placed in one renal artery under anesthesia	AD-MSCs	1.0 × 10^7^	MSCs were injected slowly through a balloon placed in the renal artery proximal to the stenosis	RBF and GFR, microvascular density, kidney histopathology, Scr, urine protein, body weight, degree of stenosis, mean arterial pressure, TNF-α, IL-10	18 weeks
4	Eirin et al.	2014	USA	Pig	Unclear	6	6	An irritant coil was placed in the main renal artery	AD-MSCs	1.0 × 10^7^	MSCs were infused into a 5F catheter engaged proximal to the stenosis	Body weight, degree of stenosis, mean arterial pressure, systolic blood pressure, diastolic blood pressure, Scr, PRA, cortical volume, cortical perfusion, RBF, GFR, TNF-α, IF-γ, MCP-1, IL-10	10 weeks
5	Zhu et al.	2015	USA	Pig	Completely randomized design	6	7	A local-irritant coil was placed in the main renal artery	AD-MSCs	2.5 × 10^5^ cells/kg	MSCs were infused into the stenotic renal artery over 5–7 min	Degree of stenosis, mean arterial pressure, PRA, creatinine, urinary protein, microvascular density, kidney histopathology, RBF, GFR, TNF-α, IL-10	10 weeks
6	Eirin et al.	2012	USA	Pig	Completely randomized design	7	7	A local-irritant coil was placed in the main renal artery in high-cholesterol pigs	AD-MSCs	1.0 × 10^7^	MSCs were injected immediately after PTRA	Body weight, degree of stenosis, mean arterial pressure, Scr, PRA, triglycerides, HDL, LDL, 8-Isoprostane, IL-1β, urinary albumin, kidney histopathology, microvascular architecture, RBF, GFR, TNF-α, IF-γ, MCP-1	16 weeks
7	Behzad et al.	2013	USA	Pig	Completely randomized design	6	7	Unilateral ARAS was induced in these pigs by placing an irritant coil in one main renal artery	AD-MSCs	1.0 × 10^7^	Animals received adipose-tissue-derived MSCs over 5 min after PTRA	Body weight, Scr, mean arterial pressure, RBF, GFR, vasa recta density, tubular injury score, VEGF, TNF-α, IL-10, MCP-1	16 weeks
8	Eirin et al.	2015	USA	Pig	Unclear	7	7	A local-irritant coil was placed in the main renal artery using fluoroscopy	AD-MSCs	1.0 × 10^7^	MSCs were injected immediately after PTRA slowly through a balloon placed in the renal artery proximal to the stenosis	Body weight, degree of stenosis, mean arterial pressure, HDL, LDL, Scr, RBF, GFR, isoprostane	16 weeks
9	Kim et al.	2020	USA	129-S1 mice	Completely randomized design	6	6	RAS was induced at baseline by surgical placement of a periarterial cuff	AD-MSCs	1.0 × 10^5^	MSCs were delivered to the RAS + MSC mice via intra-aortic injection	Body weight, kidney weight, Scr, β-galactosidase (SA-β-Gal) activity, p16, p21, p53, IL-6, TNF-α	6 weeks
10	Varela et al.	2019	Brazil	Wistar rat	Unclear	6	6	Animal model of RVH was induced by partial left renal artery obstruction	BM-MSCs	2.0 × 10^5^ (two administrations at the 3rd and 5th weeks after renal artery clipping)	MSCs were injected into the tail vein	Plasma and urinary concentrations of creatinine, urinary osmolarity, urinary flow rate, sodium excretion, APQ1, APQ2, Na/K ATPase	6 weeks
11	Oliveira-Sales et al.	2016	Brazil	Wistar rat	Unclear	9	7	The left renal artery was partially obstructed with a 0.2 mm silver clip	BM-MSCs	2.0 × 10^5^ (two administrations at the 3rd and 5th weeks after renal artery clipping)	MSCs were injected through the tail vein	Systolic blood pressure, urinary excretion of sodium, GFR, RBF, kidney weight, kidney histopathology, renin, ACE, AT1R, AT2R	6 weeks
12	Lira et al.	2016	Brazil	Wistar rat	Unclear	6	6	The left renal artery was partially obstructed with a 0.2 mm silver clip	BM-MSCs	1.0 × 10^6^	MSCs groups received only one kidney subcapsular injection	Systolic blood pressure, renin, ACE, AT_1_R, AT_2_R, Na^+^ + K^+^ ATPase activity, kidney histopathology, plasma urea, Scr, proteinuria, plasma protein	6 weeks
13	Ishiy et al.	2020	Brazil	Wistar rat	Unclear	7	7	The left renal artery was partially obstructed with a 0.2 mm silver clip	AD-MSCs	1.0 × 10^5^ (two administrations at the 3rd and 5th weeks after renal artery clipping)	MSCs were injected through the tail vein	Body weight, kidney weight, Scr, creatinine clearance, urinary volume, proteinuria, urinary sodium excretion, urinary potassium excretion, collagen type I and TGF-β, IL-1β, IL-10	6 weeks
14	Zou et al.	2018	USA	129-S1 mice	Completely randomized design	10	10	RAS was induced by surgical placement of a 0.15 mm diameter arterial cuff	AD-MSCs	1.0 × 10^5^	Carotid artery was cannulated via a vascular cut down, and MSCs were slowly injected	Systolic blood pressure, body weight, BUN, tissue oxygenation (BOLD MR), kidney volume, kidney perfusion, kidney histopathology, TGF-β, (TIMP)-1	4 weeks
15	Oliveira-Sales et al.	2013	Brazil	Wistar rat	Unclear	7	8	The left renal artery was partially obstructed with a 0.2 mm silver clip	BM-MSCs	2.0 × 10^5^ (two administrations at the 3rd and 5th weeks after renal artery clipping)	MSCs were injected into the tail vein	Systolic blood pressure, renal sympathetic nerve activity, Scr, proteinuria, body weight, plasma Na^+^ and K^+^, Urinary Na^+^ and K^+^, total urinary volume, kidney histopathology, renin, ACE, AT1R, AT2R, TNF-α, IL-10	6 weeks
16	Almeida et al.	2021	Brazil	Wistar rat	Unclear	6	8	The left renal artery was partially obstructed with a 0.2 mm silver clip	BM-MSCs	1.0 × 10^6^	MSCs were injected into the subcapsular region of the clipped kidney	Kidney histopathology, collagen IV, MMPs, TIMPs, α-SMA, IL-10	6 weeks
17	Chen et al.	2020	USA	Pig	Completely randomized design	5	5	A local-irritant coil was placed in the main renal artery in high-cholesterol pigs	AD-MSCs	1.0 × 10^7^	MSCs were injected slowly through a balloon placed in the renal artery proximal to the stenosis	Body weight, degree of stenosis, MAP, PRA, TNF-α, IF-γ, IL-10, RBF, GFR, microvascular density, capillaries-per-tubule, kidney histopathology	16 weeks

Three clinical studies, all are non-randomized controlled trials. A total of 84 patients were involved. The duration of follow-up was 3 months. A summary of the clinical studies characteristics is provided in Table 2.

**Table 2:** Characteristics of clinical studies.

	Author	Year	Country	Study design	Number of treatment group	Number of control group	MSC source	MSCs dose	Route	Endpoints	Duration	MINORS
1	Abumoawad et al.	2020	USA	Non-randomized controlled trials	19 (Low dose: 6; Medium dose: 7; High dose: 6)	18	AD-MSCs	Low dose: 1.0 × 10^5^ cells/kg; Medium dose: 2.5 × 10^5^ cells/kg; High dose: 5.0 × 10^5^ cells/kg	MSCs were administered into the renal artery of a post-stenotic kidney	eGFR, iothalamate clearance (Both kidneys), systolic blood pressure, urine protein, blood flow, Hypoxia(Cortical R2*, Fractional hypoxia), sGFR (single-kidney glomerular filtration), VEGF-A, VEGF-C, angiopoietin-2, NGAL, IFN-γ, TIMP-2	3 months	16
2	Saad et al.	2017	USA	Open-label, non-randomized controlled trials	14 (Low dose: 7; High dose: 7)	14	AD-MSCs	Low dose: 1.0 × 10^5^ cells/kg; High dose: 2.5 × 10^5^ cells/kg	Patients received single intra-arterial infusion of autologous MSCs in the renal artery	Scr, iothalamate clearance GFR, SBP, NGAL, kidney volume, cortical volume, medullary volume, cortical perfusion, medullary perfusion, RBF, Single-kidney GFR, Hypoxia (Cortical R2*, Fractional hypoxia), VEGF-C	3 months	16
3	Kim et al.	2020	USA	Non-randomized controlled trials	13	6	AD-MSCs	5.0 × 10^5^ cells/kg	Patients were treated with a single intra-arterial infusion of MSCs in the renal artery	SBP, DBP, Scr, eGFR, BMI	3 months	14

### Quality assessment

The detailed information of the quality assessment within the preclinical studies is shown in Table 3. The quality assessment results with the main characteristics of the clinical trials are listed in Table 2. The results of GRADE assessment are shown in the Table S1.

**Table 3:** Quality assessment of animal intervention studies.

	Study	Random sequence generation	Baseline characteristics	Allocation concealment	Random housing	Blinding (study team)	Random outcome assessment	Blinding (outcome assessors)	Incomplete outcome data	Selective outcome reporting	Other source of bias
1	Eirin 2015	?	?	?	?	?	?	?	+	+	+
2	Zhu 2013	?	+	?	?	?	?	?	+	+	+
3	Eirin 2014	?	?	?	?	?	?	?	+	+	+
4	Zhu 2015	?	?	?	?	?	?	?	+	+	+
5	Eirin 2012	?	?	?	?	?	?	?	+	+	+
6	Behzad 2013	?	?	?	?	?	?	?	+	+	+
7	Eirin 2015	?	?	?	?	?	?	?	+	+	+
8	Kim 2020	?	?	?	?	?	?	?	+	+	+
9	Varela 2019	?	+	?	+	?	?	?	+	+	+
10	Oliveira-Sales 2016	?	+	?	+	?	?	?	+	+	+
11	Lira 2016	?	+	?	+	?	?	?	+	+	+
12	Ishiy 2020	?	+	?	+	?	?	?	?	+	+
13	Zou 2018	?	+	?	?	?	?	?	+	+	+
14	Oliveira-Sales 2013	?	+	?	+	?	?	?	+	+	+
15	Almeida 2021	?	?	?	+	?	?	?	+	?	+
16	Chen 2020	?	?	?	?	?	?	?	+	+	+

+: low risk of bias; −: high risk of bias; ?: unclear risk of bias.

### Preclinical outcomes

#### Systolic blood pressure

**Figure 2:**
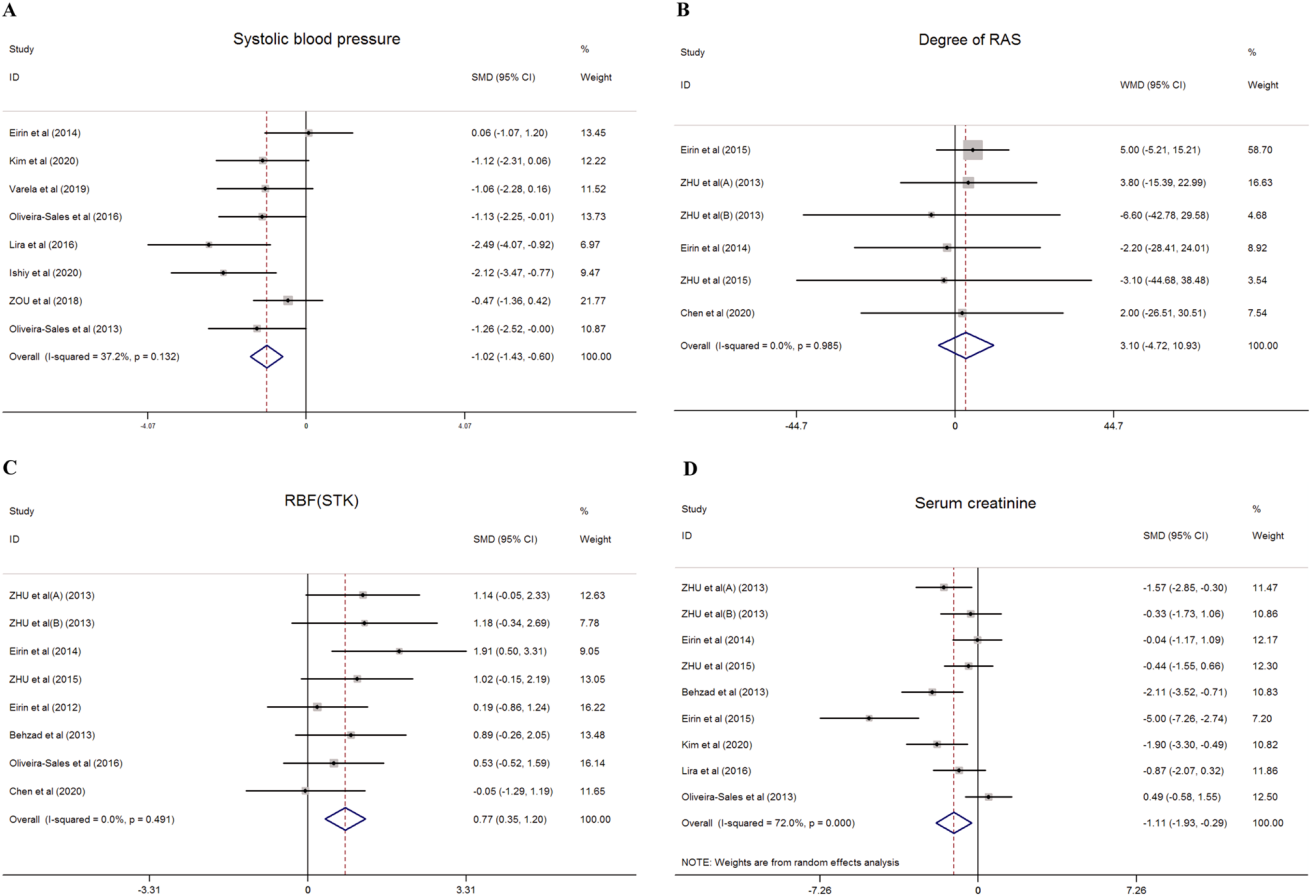
The effect of MSC therapy on systolic blood pressure (**A**), the degree of renal artery stenosis (**B**), renal blood flow of stenotic kidney (**C**) and serum creatinine (**D**) in preclinical studies.

A total of 8 studies^26,31–37^ reported systolic blood pressure (SBP) levels. A fixed-effect model using Cohen’s analysis was employed to compare the levels between the MSC treatment and control groups. Compared to the control group, the SBP decreased significantly in the MSC treatment group (SMD = − 1.019, 95% CI − 1.434 to − 0.604, I^2^ = 37.2%, P = 0.000) (Fig. 2A).

#### Mean arterial pressure

The mean arterial pressure (MAP) level was found in 9 studies^24–30,39^. We used a fixed-effect model with no standard method to compare the MSC treatment and control groups. No significant difference was observed in mean arterial pressure between the MSC treatment and control groups (SMD = − 0.751, 95% CI − 5.075 to 3.573, I^2^ = 0%, P = 0.734) (Additional file 1: Fig. S2A).

#### The degree of RAS

Six preclinical studies^24–27,39^ evaluated the degree of RAS and the outcomes demonstrated that the MSC treatment group had an equal degree of RAS compared to the control group (WMD = 3.103%, 95% CI − 4.723% to 10.928%, I^2^ = 0%, P = 0.437) (Fig. 2B). Due to low heterogeneity, a fixed-effect model was used.

#### Renal blood flow

The level of renal blood flow (RBF) in stenotic kidney (STK) was assessed in eight preclinical studies^25–29,33,39^. The pooled outcomes revealed that MSCs could increase the level of renal blood flow (SMD = 0.774, 95% CI − 0.351 to 1.197, I^2^ = 0%, P = 0.000) (Fig. 2C). A fixed-effect model was used to pool the effects of MSC administration on renal blood flow. In two studies^24,33^ RBF was detected in the contralateral kidney (CLK); no significant increase in renal blood flow was observed in the MSC treatment group (SMD = 0.518, 95% CI − 0.244 to 1.279, I^2^ = 31%, P = 0.183). In addition, significantly increased cortical perfusion was measured in 2 studies^26,36^; however, increased cortical perfusion was not observed in the MSC treatment group (SMD = 1.211, 95% CI − 0.359 to 2.781, I^2^ = 70.6%, P = 0.130).

#### Serum creatinine

The pooled results of nine studies^25–27,29–31,34,37^ measuring serum creatinine suggested that animals in the MSC group had a lower serum creatinine level (SMD = − 1.112, 95% CI − 1.932 to − 0.293, I^2^ = 72.0%, P = 0.008) (Fig. 2D). The random-effect model was employed in the analysis of serum creatinine. However, MSCs were ineffective in reducing the plasma urea nitrogen according to the pooled outcome of the 2 studies (SMD = − 0.881, 95% CI − 2.957 to 1.194, I^2^ = 83.6%, P = 0.405)^34,36^.

#### Plasma renin activity (PRA)

**Figure 3:**
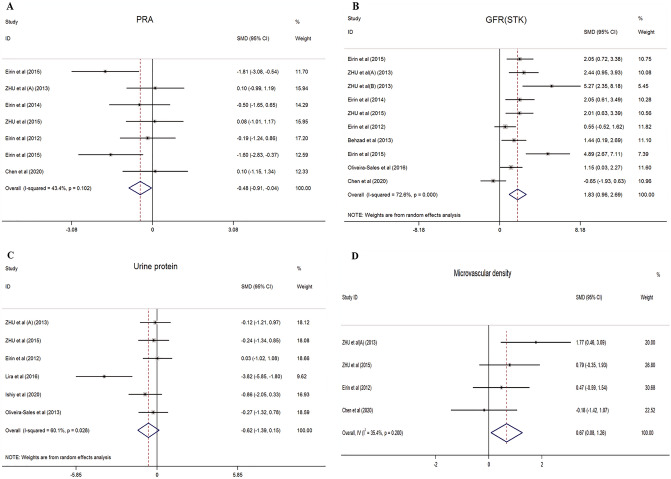
The effect of MSC therapy on plasma renin activity (**A**), glomerular filtration rate of stenotic kidney (**B**), urine protein (**C**) and microvascular density (**D**) in preclinical studies.

The plasma renin activity was measured in seven studies^24–28,30,39^. The blood samples were collected from the inferior vena cava. Compared to the control groups, the MSC administration groups had a lower level of PRA (SMD = − 0.477, 95% CI − 0.913 to − 0.042, I^2^ = 43.4%, P = 0.032) (Fig. 3A). Moreover, renin expression in STK was detected via western blot in two studies^34,37^. The expression of renin was lower in the MSC groups than in the control groups (WMD = − 0.675, 95% CI − 1.317 to − 0.033, I^2^ = 93%, P = 0.039).

#### Glomerular filtration rate

Glomerular filtration rate (GFR) in STK was measured in ten animal studies^24–30,33,39^. Compared to the control group, MSC administration preserved GFR of STK (SMD = 1.825, 95% CI 0.963 to 2.688, I^2^ = 72.6%, P = 0.000) (Fig. 3B). A random-effect model was employed due to the high heterogeneity. However, MSC administration had little benefit on GFR in CLK according to the pooled outcome of the two studies (SMD = 0.608, 95% CI − 0.149 to 1.365, I^2^ = 0%, P = 0.116)^24,33^. In addition, creatinine clearance was assessed in two studies^32,35^; however, it was not significantly different between the MSC and control groups (SMD = 0.085, 95% CI − 0.684 to 0.855, I^2^ = 0%, P = 0.828).

#### Urinary volume

Two studies^35,37^ included the assessment of urinary volume. There is no significant difference between the MSC and the control groups (WMD = − 4.835 ml, 95% CI − 21.963 ml to 12.293 ml, I^2^ = 52.7%, P = 0.580). Similarly, the urinary flow in STK (2 studies were included)^32,33^ had no significant response to MSC intervention (WMD = 0.003 ml/min, 95% CI − 0.001 ml/min to 0.006 ml/min, I^2^ = 0%, P = 0.115). Nevertheless, a higher level of urinary sodium excretion (3 studies included)^32,35,37^ and a lower level of urinary potassium excretion (2 studies included)^35,37^ were observed in the MSC group (Urinary sodium excretion: WMD = 1.486 mmol/L/min, 95% CI 0.949 mmol/L/min to 2.022 mmol/L/min, I^2^ = 0%, P = 0.000; Urinary potassium excretion: WMD = − 2.426 meq/24 h, 95% CI − 4.033 to − 0.819 meq/24 h, I^2^ = 0%, P = 0.003).

#### Urine protein

Urine protein levels were detected in six studies^25,27,28,34,35,37^. No significant difference between the MSC treatment and the control groups was observed (SMD = − 0.624, 95% CI − 1.394 to 0.146, I^2^ = 60.1%, P = 0.112) (Fig. 3C).

#### Components of the renin–angiotensin system

Furthermore, the protein levels of renin, angiotensin-converting enzyme (ACE), and Ang II receptors types1 (AT 1) and 2 (AT 2) in STK were detected using western bolt in two studies^34,37^. The MSC groups had a lower level of AT 1 receptors and a higher level of AT 2 receptors. No significant differences in ACE were observed between the MSC and the control groups (ACE: WMD = − 0.437, 95% CI − 1.128 to 0.255, I^2^ = 0%, P = 0.216; AT1R: WMD = − 0.369, 95% CI − 0.629 to − 0.109, I^2^ = 0%, P = 0.005; AT2R: WMD = 0.413, 95% CI 0.170 to 0.656, I^2^ = 0%, P = 0.001).

#### Body weight

Twelve studies^24–26,28–31,35–37,39^ assessed the level of body weight. The pooled outcomes were analyzed using a fixed-effect model. However, no apparent differences were found between the MSC and the control groups (SMD = 0.063, 95% CI − 0.256 to 0.382, I^2^ = 0%, P = 0.7) (Additional file 1: Fig. S2B).

#### Kidney weight

The kidney weight of STK was evaluated in three preclinical studies^31,33,35^. There was no significant difference between the MSC and the control groups (SMD = 0.368, 95% CI − 0.268 to 1.004, I^2^ = 30.9%, P = 0.256). The pooled effects of MSC intervention on kidney weight of CLK were also detected in three studies. Similarly, the MSC group had an equal kidney weight of CLK compared with the control group (SMD = 0.660, 95% CI − 0.573 to 1.892, I^2^ = 68.3%, P = 0.294). Besides, the volume of the cortex in STK was also measured in two studies^26,29^. The results indicated that the MSC group obtained a greater cortex volume in STK (SMD = 1.232, 95% CI 0.362 to 2.102, I^2^ = 0%, P = 0.006). However, one study showed that the volume of all STK was unaffected by MSC treatment (P > 0.05).

#### Microvascular density

Four studies^25,27,28,39^ evaluated the level of cortex microvascular density in STK using micro-CT. The pooled outcomes demonstrated that MSCs could improve cortical microvascular density in STK (SMD = 0.672, 95% CI 0.082 to 1.262, I^2^ = 35.4%, P = 0.026) (Fig. 3D). Moreover, two studies further indicated that MSC therapy was more effective in the outer cortex than in the inner cortex. Only one study assessed medullary microvascular density in STK using micro-CT, revealing that MSC intervention improves this density (P < 0.05). Besides, microvascular density in STK was also detected by CD31^28,31,36^ and vWF^28,29,39^ staining. The results showed the benefits of MSC treatment (CD31 staining: SMD = 0.672, 95% CI 0.419 to 1.684, I^2^ = 31.1%, P = 0.001; vWF staining: SMD = 1.665, 95% CI 0.314 to 3.016, I^2^ = 63.7%, P = 0.016). The number of capillaries per tubule in the cortex and medulla was measured in 3 studies^29,37,39^ showing that MSCs improve capillary density in the cortex and the medulla (Medulla: SMD = 1.186, 95% CI − 0.354 to 2.726, I^2^ = 76.7%, P = 0.131; Cortex: SMD = 1.425, 95% CI 0.518 to 2.332, I^2^ = 0%, P = 0.002).

#### Cholesterol

Total cholesterol was detected in 3 studies^28–30^. Compared to the control group, total cholesterol in MSC group has no remarkable difference. (WMD = − 18.161 mg/dl, 95% CI − 40.587 mg/dl to 4.265 mg/dl, I^2^ = 0%, P = 0.112). The levels of triglycerides, HDL (high density lipoprotein) and LDL (low density lipoprotein) were detected in two studies^28,30^. There was no significant difference in triglycerides and HDL between MSC group and control group, while MSC therapy can significantly decrease the level of LDL (Triglycerides: WMD = 2.230 mg/dl, 95% CI − 0.333 mg/dl to 4.792 mg/dl, I^2^ = 0%, P = 0.088; HDL: WMD = − 10.894 mg/dl, 95% CI − 26.536 mg/dl to 4.748 mg/dl, I^2^ = 0%, P = 0.172; LDL: WMD = − 28.243 mg/dl, 95% CI − 52.141 to − 4.345 mg/dl, I^2^ = 0%, P = 0.021).

#### Renal oxygenation

Renal oxygenation and tubular function were evaluated using blood-oxygen-level-dependent magnetic resonance imaging (BOLD MRI). The cortical and medullary oxygenation was assessed in 2^36,39^ and 3^29,36,39^ studies, respectively. The results indicated that STK cortical and medullary hypoxia was not significantly alleviated by MSC intervention (Medulla: SMD = 0.137, 95% CI − 1.537 to 1.811, I^2^ = 84.1%, P = 0.872; Cortex: SMD = 0.010, 95% CI − 0.718 to 0.738, I^2^ = 40.9%, P = 0.979). Similarly, the MSC therapy do not improve tubular function in STK (SMD = − 1.924, 95% CI − 3.998 to 0.150, I^2^ = 0%, P = 0.069)^27,29^.

#### Cytokines

The levels of net renal release of interferon-γ (IFN-γ), tumor necrosis factor-α (TNF-α), interleukin-10 (IL-10), and monocyte chemoattractant protein-1 (MCP-1) were detected. Two studies^24,26^ for IFN-γ, two studies^24,26^ for TNF-α, three studies^24,26,30^ for IL-10, and two studies^24,26^ for MCP-1 were utilized in order to evaluate the efficacy of MSCs. The pooled outcomes showed that MSCs were effective for the net renal release of IFN-γ, TNF-α, IL-10, and MCP-1 (IFN-γ: WMD = − 26.046 pg/min, 95% CI − 45.505 to − 6.587 pg/min, I^2^ = 64.1%, P = 0.009; TNF-α: WMD = − 4800 pg/min, 95% CI − 5900 to − 3800 pg/min, I^2^ = 26.2%, P = 0.000; IL-10: SMD = 2.562, 95% CI 1.676 to 3.448, I^2^ = 22.9%, P = 0.000; MCP-1: WMD = − 8200 pg/min, 95% CI − 9700 to − 6700 pg/min, I^2^ = 0%, P = 0.000) (Additional file 1: Fig. S3).

The western bolt was used to measure the levels of IFN-γ (2 studies included)^26,28^, TNF-α (7 studies included)^25–29,31,38^, IL-10 (4 studies included)^25–27,29^, MCP-1 (3 studies included)^26,28,39^, vascular endothelial growth factor (VEGF) (3 studies included)^25,28,29^, transforming growth factor-β (TGF-β) (2 studies included)^34,36^, tissue inhibitor of metalloproteinase-1 (TIMP-1) (2 studies included)^36,38^, and matrix metalloproteinase-2 (MMP-2) (2 studies included)^29,38^. In summary, the MSC group showed statistically significant decreases in the levels of IFN-γ, TNF-α, and MMP-2 as well as significant increases in the levels of VEGF and IL-10 (IFN-γ: WMD = − 0.021, 95% CI − 0.032 to − 0.010, I^2^ = 0%, P = 0.000; TNF-α: SMD = − 1.267, 95% CI − 2.163 to − 0.370, I^2^ = 70.6%, P = 0.006; MMP-2: SMD = − 1.015, 95% CI − 1.838 to − 0.192, I^2^ = 2.6%, P = 0.016; IL-10: WMD = 0.255, 95% CI − 0.132 to 0.642, I^2^ = 99.2%, P = 0.197; VEGF: WMD = 0.042, 95% CI 0.013 to 0.071, I^2^ = 0%, P = 0.005). However, there was no significant difference between the MSC and the control groups regarding the levels of MCP-1, TGF-β, and TIMP-1 (MCP-1: WMD = − 0.075, 95% CI − 0.195 to 0.044, I^2^ = 95.3%, P = 0.214; TGF-β: SMD = − 1.160, 95% CI − 3.072 to 0.752, I^2^ = 79.6%, P = 0.234; TIMP-1: SMD = 1.641, 95% CI − 1.738 to 5.021, I^2^ = 91.7%, P = 0.341).

Two studies^28,30^ were included for the measurement of circulating levels of isoprostane. The pooled outcomes suggested that MSC intervention can reduce the circulating levels of isoprostane (WMD = − 81.016, 95% CI − 99.520 to − 62.511, I^2^ = 0%, P = 0.000).

#### Renal fibrosis

Renal fibrosis was evaluated using a trichrome staining method (6 studies included)^25–28,36,39^ and a picrosirius red staining method (3 studies included)^31,34,37^. Compared with the control group, the degree of renal fibrosis was alleviated by MSC treatment (Trichrome staining: SMD = − 1.829, 95% CI − 3.002 to − 0.657, I^2^ = 77%, P = 0.002; Picrosirius red staining: SMD = − 1.444, 95% CI − 2.169 to − 0.718, I^2^ = 0%, P = 0.000).In addition, two studies^26,29^ were included to assess the tubular injury score. The MSC group had a lower score than the control group (SMD = − 3.226, 95% CI − 5.956 to − 0.496, I^2^ = 0%, P = 0.021). The percentage of glomerulosclerosis was analyzed in two studies^27,28^, and the results showed that MSC could improve glomerulosclerosis (WMD = − 2.975, 95% CI − 4.556 to − 1.394, I^2^ = 0%, P = 0.000). The apoptosis and oxidative stress levels were assessed via TUNEL^27,28,36^ and DHE^28,29,39^, respectively (TUNEL: SMD = − 1.268, 95% CI − 1.908 to − 0.629, I^2^ = 0%, P = 0.000; DHE: WMD = − 0.526, 95% CI − 1.803 to 0.752, I^2^ = 52.2%, P = 0.420).

### Clinical results

**Figure 4:**
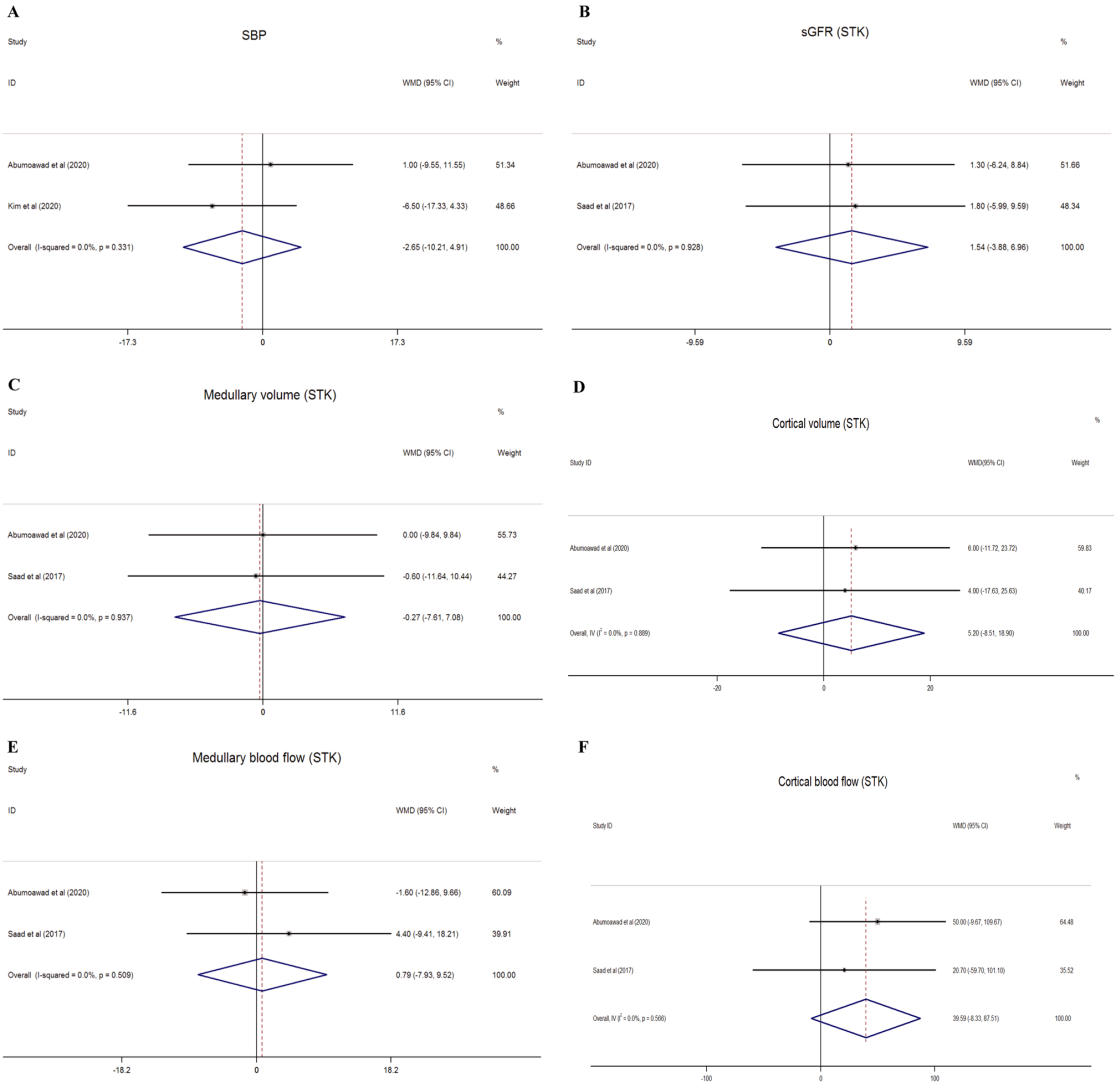
The effect of MSC therapy on systolic blood pressure (**A**), single-kidney glomerular filtration rate (**B**), medullary volume (**C**), cortical volume (**D**), medullary blood flow (**E**) and cortical blood flow (**F**) in stenotic kidney in clinical studies.

Two studies^31,40^ were included for the assessment of SBP and DBP. No benefit of MSC intervention was observed in the MSC groups (SBP: WMD = − 2.650 mmHg, 95% CI − 10.206 mmHg to 4.906 mmHg, I^2^ = 0%, P = 0.492; DBP: WMD = − 2.947 mmHg, 95% CI − 9.749 mmHg to 3.855 mmHg, I^2^ = 10.2%, P = 0.396) (Fig. 4A, Additional file 1: Fig. S4E). However, according to the results of eGFR (The Modification of Diet in Renal Disease Study, MDRD) of two kidneys in these two studies, there is no significant difference between the MSC and the control groups (WMD = − 2.590, 95% CI − 13.637 to 8.457, I^2^ = 0%, P = 0.646) (Additional file 1: Fig. S4B).

**Figure 5:**
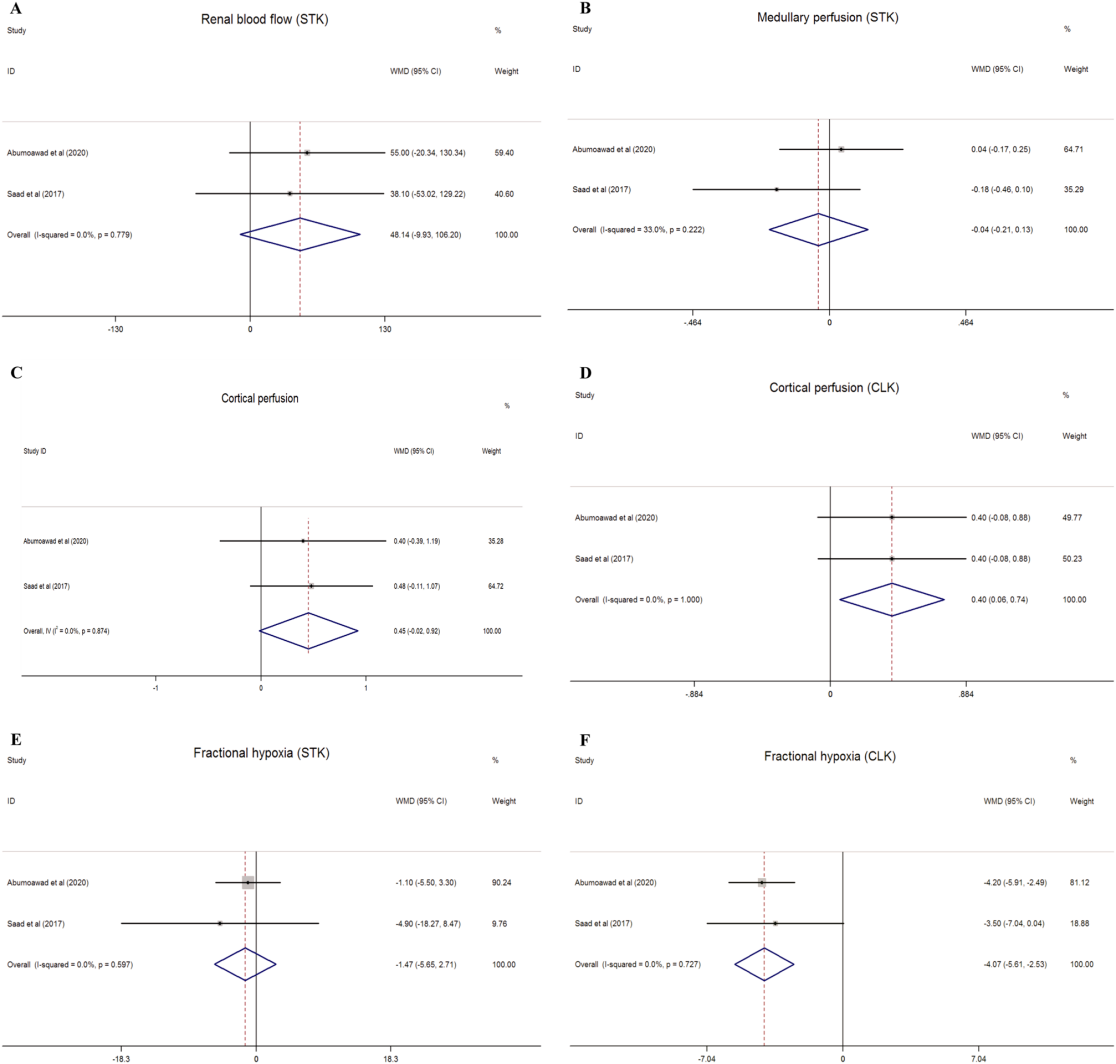
The effect of MSC therapy on renal blood flow of stenotic kidney (**A**), medullary perfusion of stenotic kidney (**B**), cortical perfusion of stenotic kidney (**C**) and contralateral kidney (**D**) as well as fractional hypoxia of stenotic kidney (**E**) and contralateral kidney (**F**) in clinical studies.

Total kidney volume, cortical volume, medullary volume, cortical perfusion, cortical blood flow, medullary perfusion, medullary blood flow, renal blood flow, and the severity of hypoxia in STK and CLK were shown in two studies^40,41^. We compared the changes from baseline to 3 months between the MSC and the medically treated groups. However, compared to the medical only treated groups, MSCs had no remarkable outcomes excepted within the cortical perfusion and fractional hypoxia of CLK (Total kidney volume of STK: WMD = 3.166 cc, 95% CI − 16.716 cc to 23.047 cc, I^2^ = 0%, P = 0.755; Total kidney volume of CLK: WMD = 5.244 cc, 95% CI − 12.768 cc to 23.255 cc, I^2^ = 0%, P = 0.568; Cortical volume of STK: WMD = 5.197 cc, 95% CI − 8.512 cc to 18.905 cc, I^2^ = 0%, P = 0.457; Cortical volume of CLK: WMD = 7.770 cc, 95% CI − 5.400 cc to 20.939 cc, I^2^ = 0%, P = 0.248; Medullary volume of STK: WMD = − 0.266 cc, 95% CI − 7.614 cc to 7.083 cc, I^2^ = 0%, P = 0.944; Medullary volume of CLK: WMD = − 2.479 cc, 95% CI − 9.723 cc to 4.766 cc, I^2^ = 0%, P = 0.502; Cortical perfusion of STK: WMD = 0.452 mL/min/cc, 95% CI − 0.019 mL/min/cc to 0.922 mL/min/cc, I^2^ = 0%, P = 0.060; Cortical perfusion of CLK: WMD = 0.400 mL/min/cc, 95% CI 0.059 mL/min/cc to 0.741 mL/min/cc, I^2^ = 0%, P = 0.022; Cortical blood flow of STK: WMD = 39.592 mL/min, 95% CI − 8.325 mL/min to 87.509 mL/min, I^2^ = 0%, P = 0.105; Cortical blood flow of CLK: WMD = 49.527 mL/min, 95% CI − 5.667 mL/min to 104.721 mL/min, I^2^ = 0%, P = 0.079; Medullary perfusion of STK: WMD = − 0.038 mL/min/cc, 95% CI − 0.206 mL/min/cc to 0.131 mL/min/cc, I^2^ = 33%, P = 0.662; Medullary perfusion of CLK: WMD = 0.011 mL/min/cc, 95% CI − 0.131 mL/min/cc to 0.153 mL/min/cc, I^2^ = 0%, P = 0.880; Medullary blood flow of STK: WMD = 0.795 mL/min, 95% CI − 7.931 mL/min to 9.520 mL/min, I^2^ = 0%, P = 0.858; Medullary blood flow of CLK: WMD = − 5.484 mL/min, 95% CI − 14.729 mL/min to 3.762 mL/min, I^2^ = 0%, P = 0.245; Renal blood flow of STK: WMD = 48.138 mL/min, 95% CI − 9.926 mL/min to 106.203 mL/min, I^2^ = 0%, P = 0.104; Renal blood flow of CLK: WMD = 46.260 mL/min, 95% CI − 14.531 mL/min to 107.050 mL/min, I^2^ = 0%, P = 0.136; Cortical R2* of STK: WMD = − 1.259 s^−1^, 95% CI − 3.241 s^−1^ to 0.722 s^−1^, I^2^ = 0%, P = 0.213; Cortical R2* of CLK: WMD = − 1.017 s^−1^, 95% CI − 2.303 s^−1^ to 0.268 s^−1^, I^2^ = 0%, P = 0.121; Fractional hypoxia % R2* > 30 of STK: WMD = − 1.471%, 95% CI − 5.647% to 2.705%, I^2^ = 0%, P = 0.490; Fractional hypoxia % R2* > 30 of CLK: WMD = − 4.068%, 95% CI − 5.606 to − 2.530%, I^2^ = 0%, P = 0.000; Single-kidney glomerular filtration of STK: WMD = 1.542 mL/min, 95% CI − 3.878 mL/min to 6.961 mL/min, I^2^ = 0%, P = 0.577; Single-kidney glomerular filtration of CLK: WMD = 0.850 mL/min, 95% CI − 5.599 mL/min to 7.300 mL/min, I^2^ = 0%, P = 0.796) (Figs. 4, 5; Additional file 1: Figs. S4, S5, S6).

### Publication bias

The funnel plots and Egger’s tests showed significant publication bias in GFR of STK (P = 0.006) (Additional file 1: Fig. S1).

## Discussion

In our meta-analysis, 16 preclinical and 3 clinical studies in 18 publications were included to evaluate the efficacy of MSC therapy for RAS. No adverse events were reported in the animal and the clinical researches. In the preclinical studies, significant differences were observed in the levels of SBP and RBF in STK, Scr, RRA, GFR, as well as in the microvascular density of the cortex. Besides, the levels of cytokines, including the net renal release of IFN-γ, TNF-α, IL-10, and MCP-1, as well as renal fibrosis, also responded to MSC therapy. The results suggest that MSC might be a potential therapeutic agent for RAS. However, similar efficacy of MSCs administration did not appear in clinical trials. Furthermore, only the cortical perfusion and fractional hypoxia of CLK were improved by MSC therapy, indicating an urgent need for larger and precise clinical trials.

In previous studies, MSCs showed an attractive potential in many other kidney and ischemic diseases. Zou et al.^42^ published a meta-analysis regarding the efficacy of MSC administration in lupus nephritis. The pooled results demonstrated that reduced ds-DNA, ANA, Scr, BUN, proteinuria, and renal sclerosis score were seen with MSC treatment. Lin et al.^43^ performed a meta-analysis to assess the efficacy of MSC treatment on diabetic kidney disease without species limitations. Their studies verified that MSC management can result in lower levels of BUN, Scr, and urinary protein, enhance the glycemic management and alleviate the renal fibrosis in animal DKD models. However, MSCs had minimal benefits in the DKD clinical trial. Lalu et al.^44^ conducted a meta-analysis regarding MSC therapy for stroke and confirmed that MSC therapy improves several neurological and motor function tests. However, poor effects of MSC therapy on stroke were observed in clinical studies. Moreover, according to the study of Wahid et al.^45^, MSC treatment made no difference in the 'no-option' critical lower limb ischemia patients. Obviously, there is a barrier in the bench to bedside translation of MSC therapy.

Our meta-analysis is the first research to evaluate the efficacy of MSC therapy in RAS. RAS gradually progresses over a long period of time. Complicated mechanisms including hemodynamic changes, tubulointerstitial hypoxia, and activation of the RAAS system are involved during the prolonged process^46,47^. Hemodynamic changes often lead to tubulointerstitial hypoxia and activation of the RAAS system. Furthermore, tubulointerstitial hypoxia and the activation of the RAAS system can trigger oxidative stress and inflammation^48^, leading to tissue injury and interstitial fibrosis. Unlike acute hypoxia, chronic hypoxia resulted in microvascular remodeling and rarefaction, further accelerating the progression of renal fibrosis and dysfunction. Previous studies have shown that MSCs play an important role in angiogenic factors release, inflammation reduction, hemodynamic stabilization, and mitigation of oxidative stress, apoptosis, microvascular rarefaction, and fibrosis^25,26,28^. In the included preclinical studies, the suppression of the renin-angiotensin system in the MSC groups was confirmed by the decreased levels of PRA and the AT1 receptor and increased levels of the AT2 receptor, leading to the reduction of SBP. The microvascular regeneration detected by the micro-CT and the increased VEGF in the MSC groups might contribute to the increased RBF despite the unchanged patency of the renal artery. The increased GFR of STK seems to benefit from the increased RBF and renal fibrosis improvement. In the included clinical studies, although poor outcomes were described previously, Abumoawad’s and Saad’s studies discovered that RBF and perfusion were improved in the medical plus the MSC groups compared to baseline. Furthermore, Abumoawad et al. found dose–response changes in the estimated glomerular filtration rate, urine protein, and diastolic blood pressure. Saad et al. discovered dose–response changes in the RBF of STK and CLK. Kim’s study found that the efficacy of MSCs is decreased in SBP. However, the efficacy of reducing SBP was not significant when pooled with the data in Abumoawad’s studies. In addition, Abumoawad et al. and Saad et al. both indicated that inflammatory markers tended to improve. Nonetheless, the efficacy of MSCs in clinical trials is still doubtful.

There are many possible reasons for the discrepancy of MSC efficacy between preclinical and clinical studies. First, the clinical trials were nonrandomized with relatively few patients and diabetic patients were excluded. Second, clinical trials were all at an early phase. The studies of Abumoawad et al. and Saad et al. are at phase 1a and phase 1/2a, respectively. The therapeutic conditions such as effective dosage of MSCs are still under-explored in the trials. Moreover, both studies ignored important clinical outcomes such as Scr, the degree of RAS and PRA. Kim et al. also performed a preliminary clinical study. Kim’s study provided deficient clinically relevant outcomes while they focused on the cellular senescence in STK. Third, dose–response changes should be evaluated. More significant changes and no adverse events were observed within the clinical outcomes when MSC doses was increased during the therapeutic intervention. Therefore, the dosage applied in the included trials might not meet the effective dosage. Proper dose escalation of MSCs should be considered in future trials. In addition, previous studies had verified that repeated MSC delivery brought benefits to rodent models^35,37^; thus, the frequency of MSC administration can also be taken into account. Fourth, the timing of MSC administration may also influence the efficacy of MSC delivery. In preclinical studies, MSCs were delivered from 2 to 6 weeks after renal artery clamping. MSC delivery was performed twice at the 3rd and 5th week within the four rat studies and once at the 6th week in all pig RAS models. MSC administrations were performed in the early stages of RAS in the preclinical studies. However, three clinical trials did not report the course of the disease. We speculated that there might be some enrolled patients in the chronic phase when the MSCs were delivered. Lerman et al.^6^ suggested that circulation of self-perpetuating tissue damage existed during RAS progression; therefore, the severity of RAS can be aggravated over time. Thus, the timing of MSC delivery as well as the course of RAS needs to be taken seriously.

According to the quality assessment results within the preclinical studies, none of the 16 articles met the ten criteria of low risk in the SYRCLE tool. Most studies only met three to five of the ten criteria. The major biases were the results of undefined random sequence generation methods, unclear allocation concealment, and the absence of the blinding of the animal breeders, researchers, and outcome assessors. The quality of the included clinical studies was at an intermediate level. The scores were deducted to exclude potential patients, lack of blinding, and calculation of the study size. Our quality assessment was limited by poor reports regarding the methodological protocols within the included articles. Thus, we sometimes had to make choices based upon our judgment. Therefore, future studies should show detailed protocols.

The study has some limitations. First, the clinical studies were non-randomized controlled trials with few patients. High-quality RCTs and more comprehensive clinical outcomes are needed for a stronger conclusion. Second, the period after MSC intervention mostly ranged from 2 to 4 weeks within the preclinical studies and was set at 3 months in the clinical studies; thus, the long-term effects cannot be observed. Third, different types of MSCs and species were used, which influenced the conclusion of the preclinical studies. Fourth, all included studies did not report adverse events. However, the study sizes were limited; therefore, more extensive studies should be performed in order to verify the safety of MSC delivery. At last, due to the relative lack of related researches, this meta-analysis included both random and non-random studies without species limitations, which can lead to low quality of evidence. Abundant high-quality studies should be conducted to pool more reliable results in the future.

## Conclusion

In our meta-analysis, we provide preliminary evidences for the MSC therapy of RAS. In preclinical studies, MSC therapy might lead to decreased levels of SBP, Scr, PRA, IFN-γ, TNF-α, and MCP-1 and increased levels of RBF, GFR, microvascular density, and IL-10. Meanwhile, renal fibrosis can be alleviated after MSC therapy. In the clinical trials, only the cortical perfusion and fractional hypoxia of CLK benefits from MSC therapy. Nevertheless, this meta-analysis demonstrates that MSC therapy might be a potential therapy for RAS treatment. Although the MSC therapy isn’t recommended in clinical care right now, the cell-based therapy should attract more clinicians’ attention. In future, more well-designed basic studies should be performed to improve study quality, determine the optimal MSC delivery and dosage, and understand the global biological mechanisms of the observed protective effects. We hope that credible basic studies can provide important clues for the design of clinical trials and promote the bedside translation of MSC therapy.

## Supplementary Information

Supplementary Information 1.

Supplementary Information 2.

## Data Availability

All support data are included in this article.

## Abbreviations

MSC: Mesenchymal stem cell
RAS: Renal artery stenosis
RVD: Renovascular disease
STK: Stenotic kidney
CLK: Contralateral kidney
WMD: Weighted mean difference
SMD: Standard mean difference
SYRCLE: Systematic Review Centre for Laboratory Animal Experimentation
MINORS: Methodological Index for Non-randomized Studies
SBP: Systolic blood pressure
DBP: Diastolic blood pressure
MAP: Mean arterial pressure
RBF: Renal blood flow
PRA: Plasma renin activity
TNF-α: Tumor necrosis factor-α
MCP-1: Monocyte chemoattractant protein-1
AD-MSCs: Adipose tissue-derived mesenchymal stem cells
BM-MSCs: Bone marrow mesenchymal stem cells
