# Supplementary material for: Effects of mesenchymal stem cells in renovascular disease of preclinical and clinical studies: a systematic review and meta-analysis
Source: Sci Rep. 2022 Oct 27;12:18080. doi: 10.1038/s41598-022-23059-2 (PMC9613984; doi:10.1038/s41598-022-23059-2)
Supplement: Supplementary file 1 — Supplementary Information 1. [file 41598_2022_23059_MOESM1_ESM.docx]

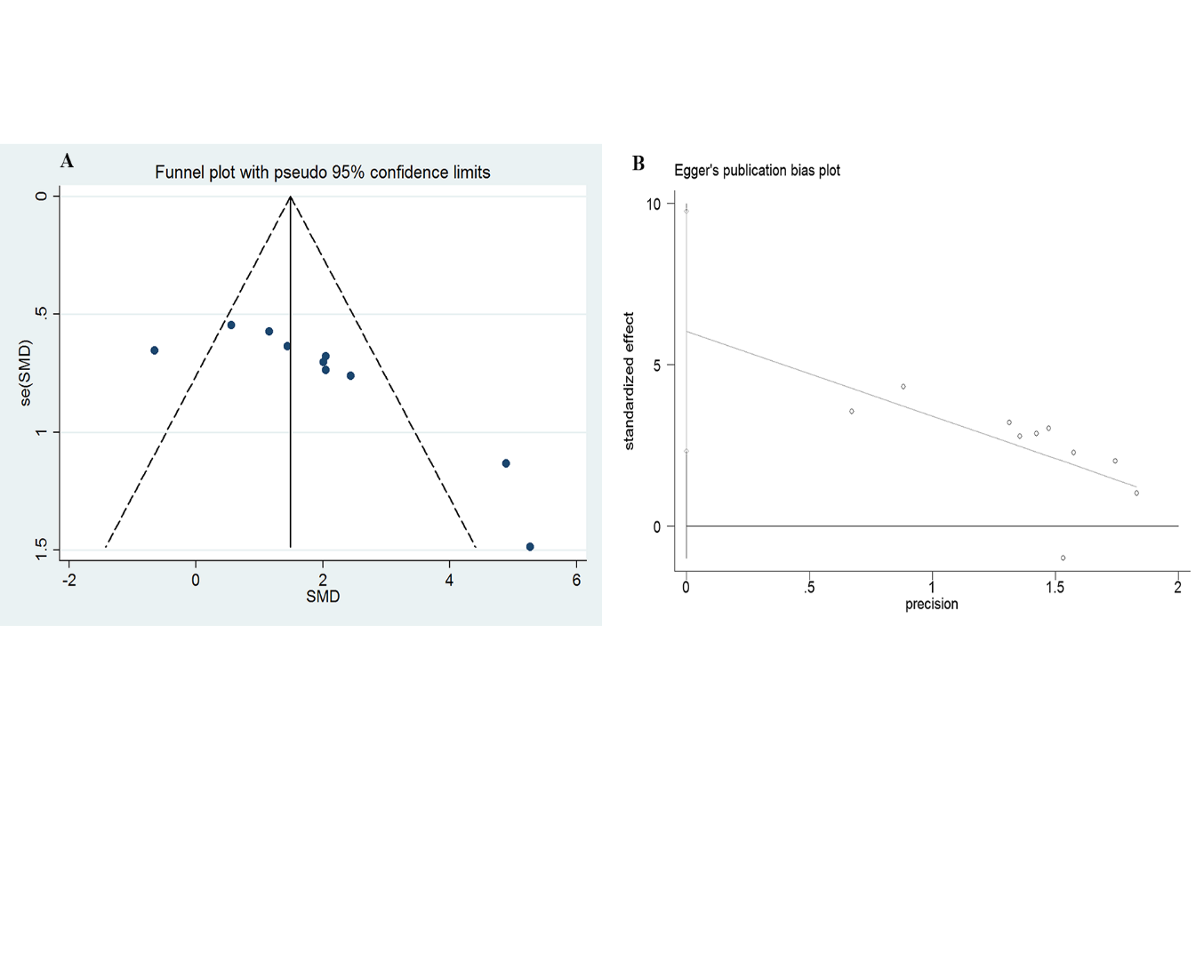


**Figure S1** Publication bias of the outcomes. (A) GFR of stenotic kidney; (B) Egger’s test for GFR;


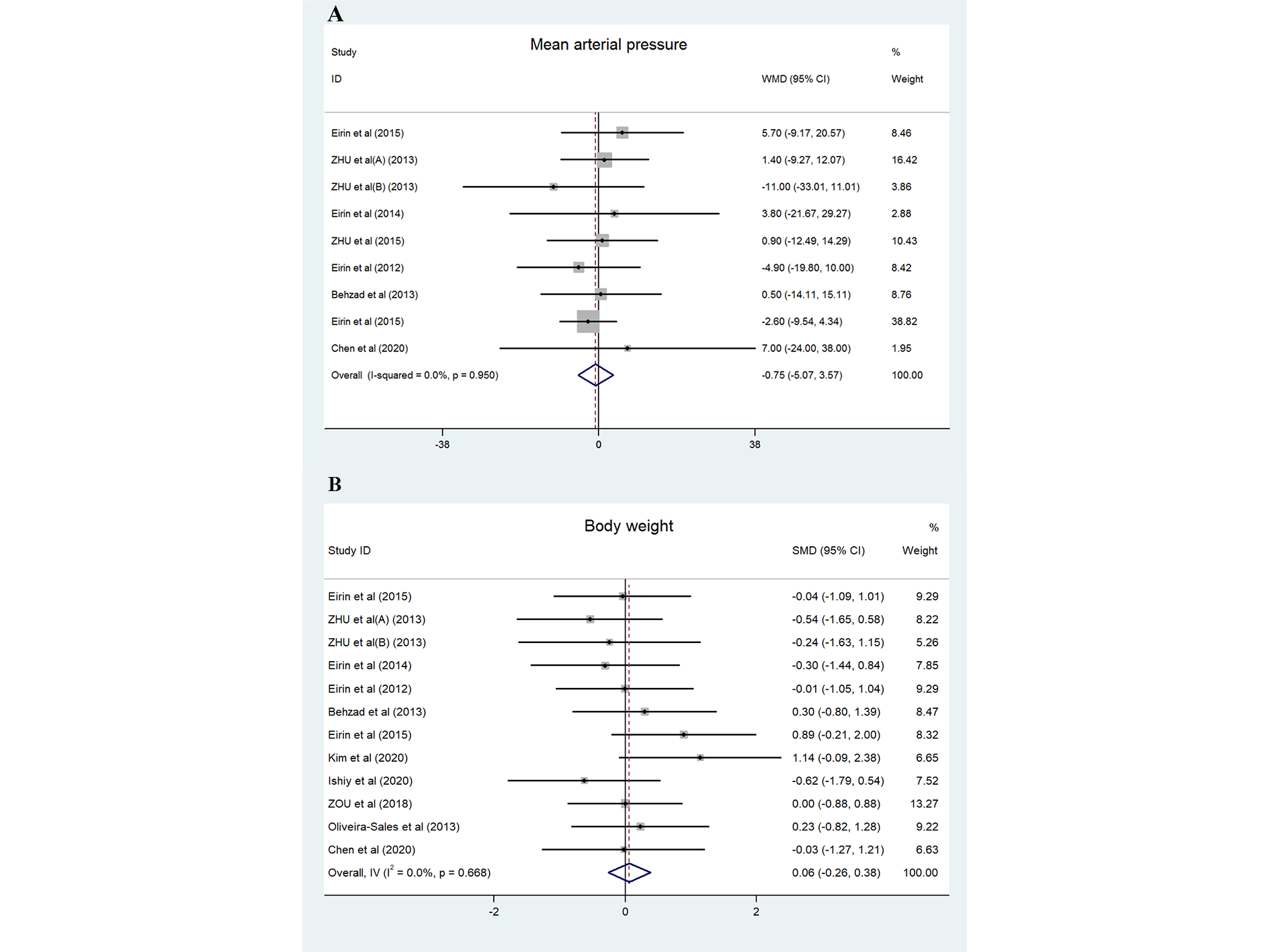


**Figure S2** The effect of MSC therapy on mean arterial pressure (A) and body weight (B) in preclinical studies.


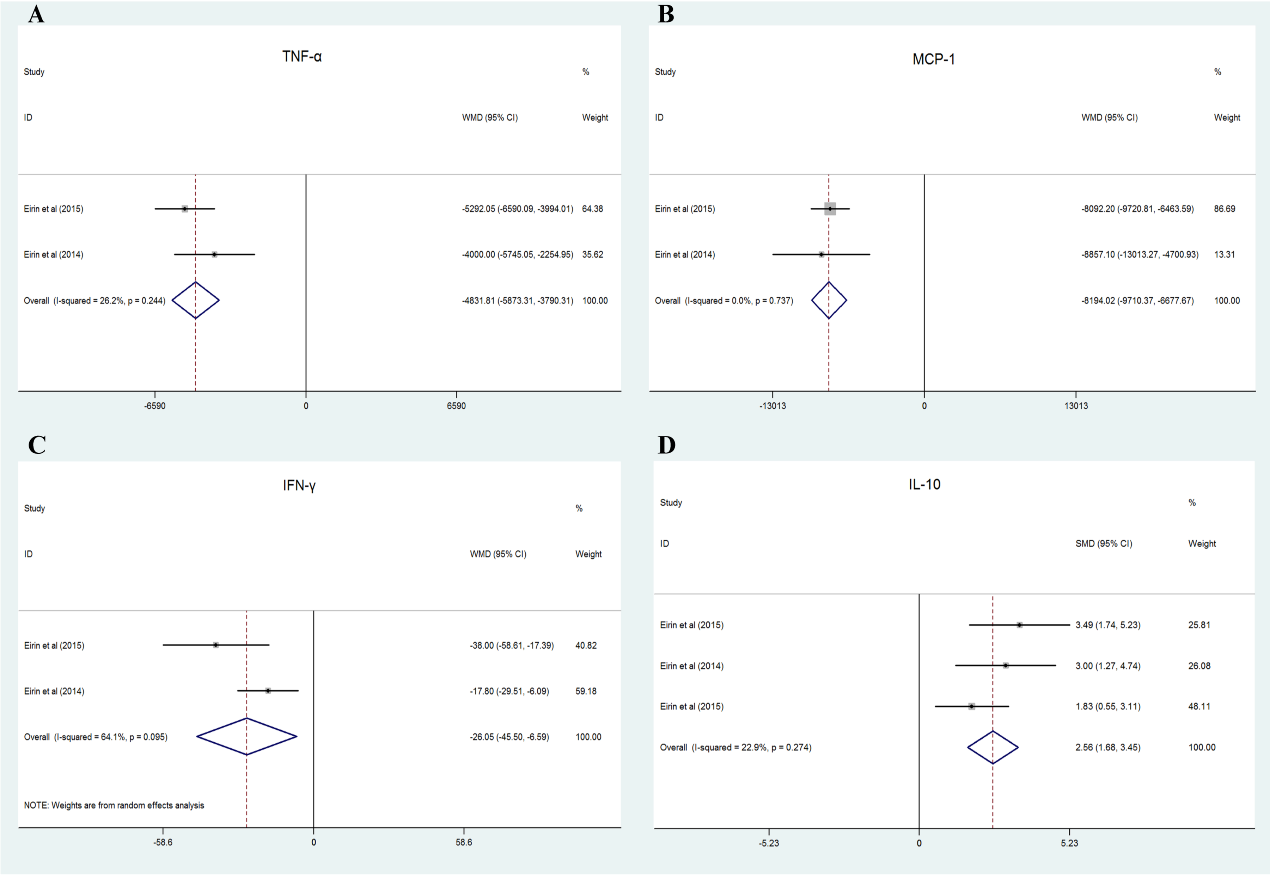


**Figure S3** The effect of MSC therapy on the net renal release of TNF-α (A), MCP-1 (B), IFN-γ (C), and IL-10 (D) in preclinical studies.


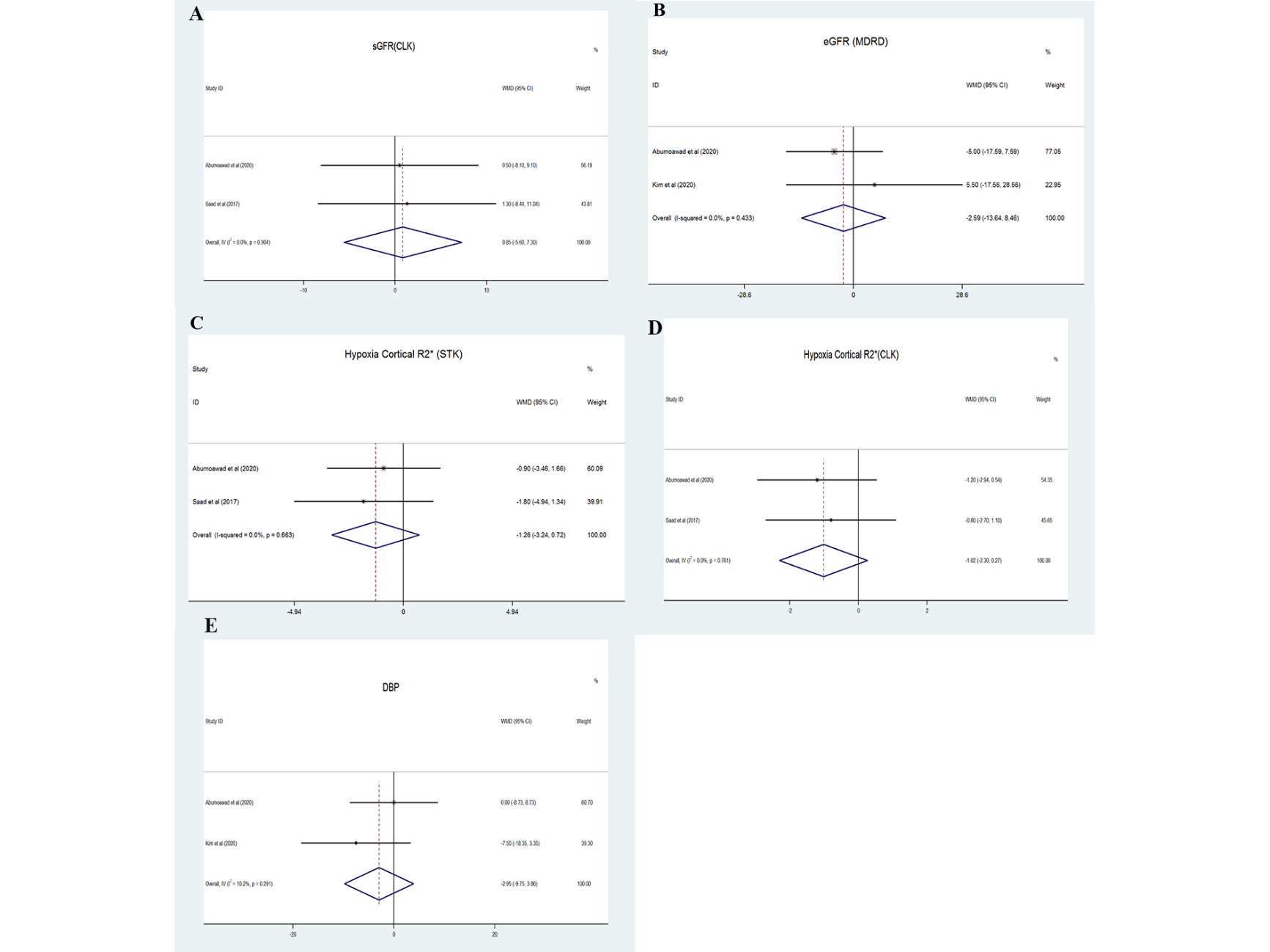


**Figure S4** The effect of MSC therapy on the sGFR of contralateral kidney (A), eGFR (MDRD) (B), hypoxia cortical R2* of stenotic kidney (C) and contralateral kidney (D) as well as diastolic blood pressure (E) in clinical trials.


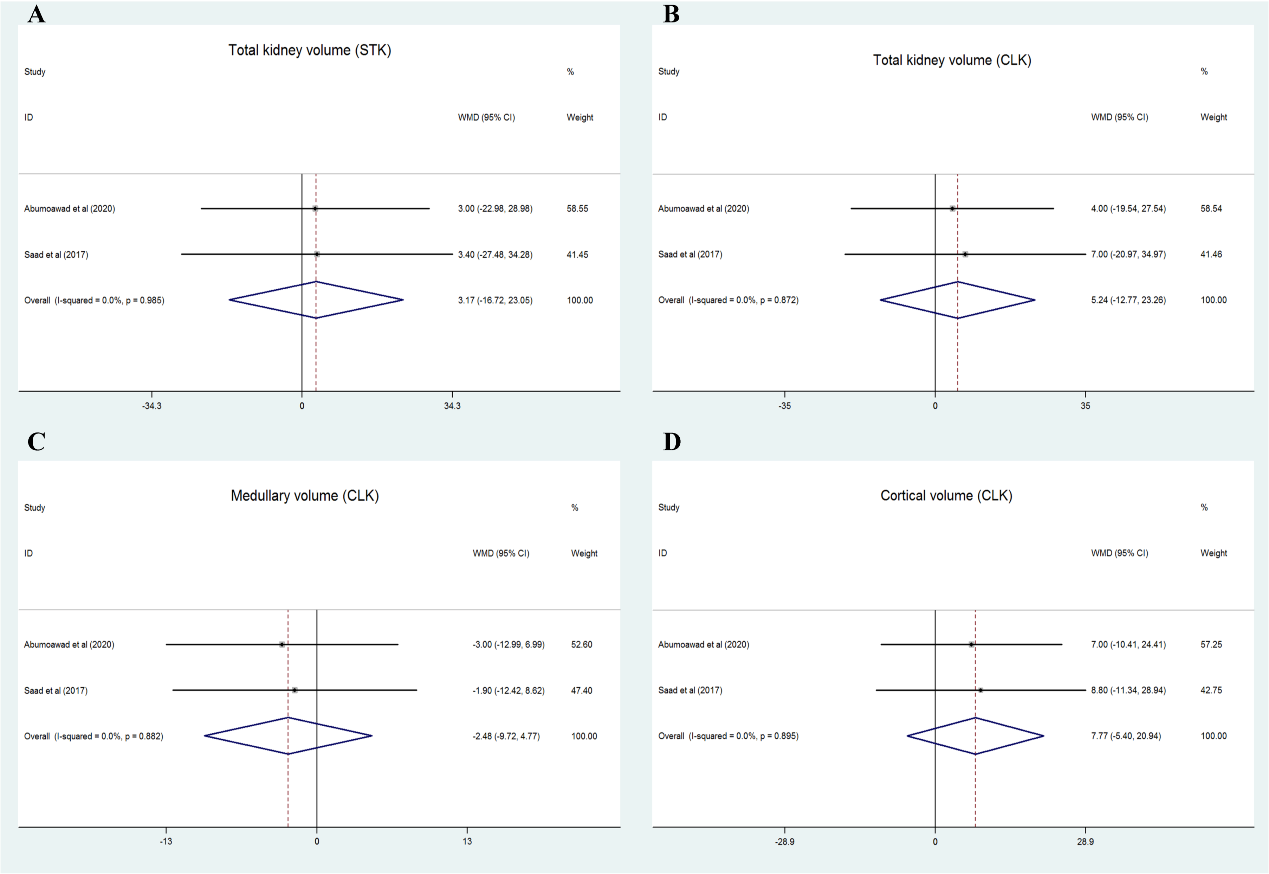


**Figure S5** The effect of MSC therapy on total kidney volume of stenotic kidney (A) and contralateral kidney (B), medullary volume of contralateral kidney (C) and cortical volume of contralateral kidney (D) in clinical trials.


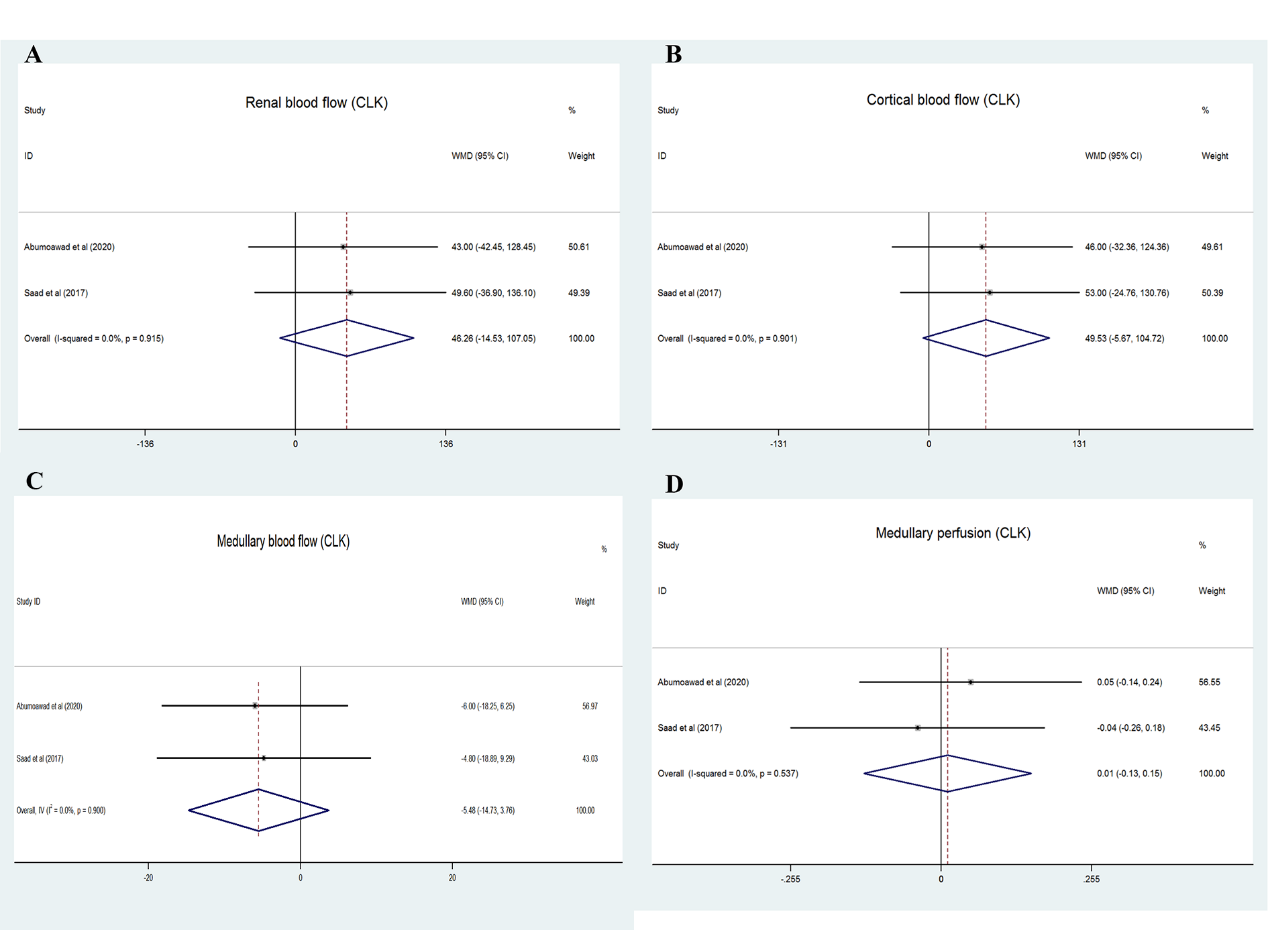


**Figure S6** The effect of MSC therapy on renal blood flow of contralateral kidney (A), cortical blood flow of contralateral kidney (B), medullary perfusion of contralateral kidney (C), and medullary blood flow (D) of contralateral kidney.

**Table S1** GRADE assessment for preclinical and Clinical outcomes

| **Preclinical outcomes** | **Quality grading** |  |
| --- | --- | --- |
|  |  |  |
| **Systolic blood pressure (SBP):** SBP of RAS animals decreases significantly after MSCs administration. | Low |  |
| **Serum creatinine (Scr):** MSC therapy can reduce the level of Scr in RAS animals. | Low |  |
| **Renal blood flow (RBF) of STK:** The level of RBF of STK significantly increases after MSCs administration. | Low |  |
| **Plasma renin activity (PRA) of STK:** MSC treatment can reduce the level of PRA of STK in RAS animals. | Low |  |
| **Glomerular filtration rate (GFR) of STK:** MSC therapy can improve the GFR of STK. | Low |  |
| **Renal fibrosis (trichrome staining):** After MSCs administration, the renal fibrosis can be alleviated. | Low |  |
| **Interferon-γ (IFN-γ):** The level of net renal release of IFN-γ decreases significantly after MSCs administration. | Very low |  |
| **Tumor necrosis factor-α (TNF-α):** The level of net renal release of TNF-α is lower after MSCs treatment. | Very low |  |
| **Interleukin-10 (IL-10):** The levels of net renal release of IL-10 significantly increases after MSCs administration. | Low |  |
| **Monocyte chemoattractant protein-1 (MCP-1):** The level of net renal release of MCP-1 decreases after MSCs treatment. | Very low |  |
| **Clinical outcomes** |  |  |
|  |  |  |
| **Cortical perfusion of CLK:** After MSC treatment, there is a significant increase in the level of cortical perfusion of CLK. | Low |  |
| **Fractional hypoxia of CLK:** MSC treatment can reduce the level of fractional hypoxia of CLK. | Low |  |
