# Supplementary material for: Effects of mesenchymal stem cells in renovascular disease of preclinical and clinical studies: a systematic review and meta-analysis
Source: Sci Rep. 2022 Oct 27;12:18080. doi: 10.1038/s41598-022-23059-2 (PMC9613984; doi:10.1038/s41598-022-23059-2)
Supplement: Supplementary file 2 — Supplementary Information 2. [file 41598_2022_23059_MOESM2_ESM.docx]

| **Section and Topic** | **Item #** | **Checklist item** | **Location where item is reported** |
| --- | --- | --- | --- |
| **TITLE** | | |  |
| Title | 1 | The report is identified as a meta-analysis. | Page 1 |
| **ABSTRACT** | | |  |
| Abstract | 2 | Report an abstract addressing each item in the PRISMA 2020 for Abstracts checklist. | Page 2 |
| **INTRODUCTION** | | |  |
| Rationale | 3 | Stated in the Introduction. | Page 3 |
| Objectives | 4 | Described in the Introduction. | Page 4 |
| **METHODS** | | |  |
| Eligibility criteria | 5 | The Eligibility criteria is described in Materials and methods. | Page 5 |
| Information sources | 6 | The information sources are described in Materials and methods. The name and URL (uniform resource locator) of each source is presented in the reference. | Page 4 |
| Search strategy | 7 | The search strategy described in Materials and methods. | Page 4 |
| Selection process | 8 | Two investigators independently reviewed the titles and abstracts according to inclusion and exclusion criteria. Once disagreements arose, a third investigator reviewed the articles and made the decision. | Page 6 |
| Data collection process | 9 | Relevant data were recorded in a standardized form in Microsoft Excel. The data were extracted from graphics using Get Data Graph Digitizer 2.25 software for studies that did not supply direct results. | Page 6 |
| Data items | 10a | The outcomes are described in Materials and methods and listed in Table 1 and Table 2. | Page 6 |
|  | 10b | Other variables (such as intervention characteristics) are listed in Table 1 and Table 2. | Table 1 and 2 |
| Study risk of bias assessment | 11 | For preclinical studies, the SYRCLE risk of bias tool was employed for quality assessment. For the clinical studies, we used the MINORS tool for the non-randomized controlled studies. | Page 6 |
| Effect measures | 12 | The effect measure is described in the Materials and methods. | Page 7 |
| Synthesis methods | 13a | The weighted mean difference (WMD) and standard mean difference (SMD) with 95% confidence intervals (CIs) were used for appropriate continuous variables. | Page 7 |
|  | 13b | All median with range or interquartile range were converted to the form mean with standard deviation. | Page 7 |
|  | 13c | The forest plot is used to display the effect estimates and confidence intervals of each study and often the summary estimate. | Figure 1-5 |
|  | 13d | Heterogeneity was assessed by I^2^ and considered significant when I^2^ >50%. STATA 12.0 statistical software package (Stata Corporation, College Station, TX) was used for statistical analysis. | Page 7 |
|  | 13e | Meta-regression is used to explore possible causes of heterogeneity. | Page 7 |
|  | 13f | Describe any sensitivity analyses conducted to assess robustness of the synthesized results. |  |
| Reporting bias assessment | 14 | Potential publication bias was assessed via Funnel plots, as well as the Bagger’s and Egger’s tests. | Page 7 |
| Certainty assessment | 15 | The quality assessment was carried out by two investigators independently, and a third investigator resolved any disagreements. | Page 6 |
| **RESULTS** | | |  |
| Study selection | 16a | The results of the search and selection process is described in Study selection and presented in a flowchart. | Page 7 |
|  | 16b | Some studies that might appear to meet the inclusion criteria are excluded due to lack of data, being off-topic, and unavailability of full texts. | Page 7 |
| Study characteristics | 17 | The study characteristics are presented in Study characteristic, Table 1 and Table 2. | Page 8 |
| Risk of bias in studies | 18 | The detailed information of the quality assessment within the preclinical studies is shown in Table 3. The quality assessment results with the main characteristics of the clinical trials are listed in Table 2. | Page 8 |
| Results of individual studies | 19 | All outcomes are presented in the Preclinical outcomes and Clinical outcomes. | Page 9 and Page 18 |
| Results of syntheses | 20a | The characteristics and risk of bias among contributing studies are presented in Study characteristic, Table 1 and Table 2. | Page 8 |
|  | 20b | Results of all statistical syntheses are presented in the Preclinical outcomes and Clinical outcomes. | Page 9 and Page 18 |
|  | 20c | Results of all investigations of possible causes of heterogeneity are presented in the Preclinical outcomes and Clinical outcomes. | Page 9 and page 18 |
|  | 20d | The effects of the outcomes were pooled using a fixed-effect model, while a random model was employed when significant heterogeneity was detected. | Page 7 |
| Reporting biases | 21 | Potential publication bias was assessed via Funnel plots, as well as the Bagger’s and Egger’s tests. | Page 20 |
| Certainty of evidence | 22 | Assessments of certainty in the body of evidence for each outcome assessed are presented in the Preclinical outcomes. | Page 9 |
| **DISCUSSION** | | |  |
| Discussion | 23a | A general interpretation of the results in the context of other evidence was provided in Discussion. | Page 21 |
|  | 23b | Discuss any limitations of the evidence included in the review. | Page 22 |
|  | 23c | Discuss any limitations of the review processes used. | Page 24 and Page 26 |
|  | 23d | Discuss implications of the results for practice, policy, and future research. | Page 25 |
| **OTHER INFORMATION** | | |  |
| Registration and protocol | 24a | Registration dose not apply. |  |
|  | 24b | The protocol was not prepared. |  |
|  | 24c | No amendment is applied. |  |
| Support | 25 | Describe sources of financial or non-financial support for the review, and the role of the funders or sponsors in the review. |  |
| Competing interests | 26 | The authors declare no competing interests. | Page 29 |
| Availability of data, code and other materials | 27 | All support data are included in this article. | Page 28 |

*From:*  Page MJ, McKenzie JE, Bossuyt PM, Boutron I, Hoffmann TC, Mulrow CD, et al. The PRISMA 2020 statement: an updated guideline for reporting systematic reviews. BMJ 2021;372:n71. doi: 10.1136/bmj.n71

For more information, visit: <http://www.prisma-statement.org/>
